# Inverted internal limiting membrane insertion combined with air tamponade in the treatment of macular hole retinal detachment in high myopia: study protocol for a randomized controlled clinical trial

**DOI:** 10.1186/s13063-018-2833-y

**Published:** 2018-08-30

**Authors:** Ying Zheng, Mei Kang, Hong Wang, Haiyun Liu, Tao Sun, Xiaodong Sun, Fenghua Wang

**Affiliations:** 1 Department of Ophthalmology, Shanghai General Hospital, Shanghai Jiaotong University School of Medicine, Building 1, No.100, Haining Road, Shanghai, 200080 China; 2 Clinical Research Center, Shanghai General Hospital, Shanghai Jiaotong University School of Medicine, Shanghai, China

**Keywords:** Macular hole retinal detachment, High myopia, Inverted internal limiting membrane insertion, Air tamponade

## Abstract

**Background:**

Macular hole retinal detachment (MHRD) occurs most commonly in high myopia and causes severe visual impairment and greatly reduces the quality of life. The aim of this study is to evaluate the efficacy and safety of inverted internal limiting membrane insertion combined with air tamponade in the treatment of MHRD in high myopia, and also to compare the treatment efficacy with that of the conventional “vitrectomy plus internal limiting membrane peeling plus silicone oil tamponade” method for high myopia-associated MHRD.

**Methods/design:**

In this clinical trial, 38 patients with MHRD in high myopia will be randomly assigned to two groups (Group 1: standard 3-port 23-gauge pars plana vitrectomy plus internal limiting membrane peeling plus air-fluid exchange plus silicone oil infusion; Group 2: standard 3-port 23-gauge pars plana vitrectomy plus internal limiting membrane peeling plus inverted internal limiting membrane insertion plus air-fluid exchange). The primary outcome is macular hole closure rate in 3 months after the initial surgery. The secondary outcomes are best corrected visual acuity (BCVA), reattachment rate of retinal detachment, and postoperative complication rate.

**Discussion:**

The study results may help to evaluate the efficacy and safety of inverted internal limiting membrane insertion combined with air tamponade in the treatment of MHRD in high myopia, and also compare the efficacy of the new treatment with the conventional “vitrectomy plus internal limiting membrane peeling plus silicone oil tamponade” method. This trial may provide a novel surgical treatment for MHRD in high myopia with more effectiveness and less pain.

**Trial registration:**

ClinicalTrials.gov, NCT03383731. Registered on 19 December 2017. Retrospectively registered.

**Electronic supplementary material:**

The online version of this article (10.1186/s13063-018-2833-y) contains supplementary material, which is available to authorized users.

## Background

Macular hole retinal detachment (MHRD) occurs most commonly in high myopia and causes severe visual impairment and greatly reduces the quality of life [1, 2]. Studies show that it accounts for about 0.5–5% of rhegmatogenous retinal detachment worldwide and about 9–21% in China [3, 4]. The possible mechanisms are tangential traction on the retina by an epiretinal membrane, posterior enlargement of the staphyloma, and atrophy of the retinal pigment epithelium [5]. With the increasing number of patients with myopia, the number of cases of MHRD resulting from high myopia has also risen dramatically. Therefore, treatment for high myopia-associated MHRD is not only a medical issue, but also a public health concern.

The conventional treatments include gas injection [6], macular buckling, and scleral imbrication [7], which have a high rate of postoperative complications and require more surgeries to achieve anatomic reattachment. Recent studies demonstrated that pars plana vitrectomy with intraocular tamponade achieve satisfactory surgical outcomes [8]. A multicenter, prospective, randomized and controlled clinical trial showed that the key factor for success of the MHRD surgery is to remove the vitreous cortex, and the internal limiting membrane (ILM) dyeing technique assists with the complete removal of the posterior hyaloid [9]. Thus, vitrectomy combined with ILM peeling plus expansive gas or silicone oil tamponade is currently the main treatment for MHRD in high myopia.

Although ILM peeling has increased the rate of retinal reattachment, the closure rate of macular holes ranged from 10 to 70% in MHRD [10–16]. In an idiopathic macular hole, ILM peeling and inverted ILM insertion can increase the closure rate of the macular hole (up to 98%). After the operation, the diameter of the ellipsoid zone defect is significantly reduced, and visual acuity also improves significantly [17–19]. Therefore, vitrectomy combined with ILM peeling plus inverted ILM insertion is gradually becoming the mainstream method for the treatment of idiopathic macular holes.

The current standard intraocular tamponade, which includes silicone oil and an expansive gas, such as C_3_F_8_ or SF_6_, sustains a very long time, and during the duration, retinal detachment seldom recurs. However, the use of silicone oil and expansive gas can lead to postoperative complications such as elevated intraocular pressure, rapid cataract progression, and corneal degeneration. The use of sterilized air is the third kind of intraocular tamponade. It sustains for a shorter time and can bring early exposure to the macular region, shorten postoperative prone time, reduce pain, and also can reduce operation time and cut-off procedures for the infusion of silicone oil or expansive gas, which can thereby reduce the risk of surgical infection [20]. Compared with silicone oil infusion, sterilized air tamponade has the same repair effect in patients with an idiopathic macular hole and primary retinal detachment caused by a peripheral retinal hole [21, 22].

Based on the above, we hypothesized that vitrectomy plus ILM peeling plus inverted ILM insertion plus air tamponade might be an effective and safe method to increase the macular hole closure rate, reduce the retinal redetachment rate, and improve visual acuity of patients with MHRD and high myopia.

Therefore, the aim of this study is to evaluate the efficacy and safety of inverted ILM insertion combined with air tamponade in the treatment of MHRD in patients with high myopia, and also to compare the treatment efficacy between different surgical approaches for MHRD.

## Methods/design

### Study aim

The aim of this study is to evaluate the efficacy and safety of inverted ILM insertion combined with air tamponade in the treatment of MHRD in patients with high myopia, and also to compare the treatment efficacy with that of the conventional “vitrectomy plus ILM peeling plus silicone oil tamponade” method for high myopia-associated MHRD.

### Organization

The principal investigator (PI) is responsible for the overall project and for organizing steering committee meetings. An independent steering committee will be responsible for ensuring the overall safety of participants, coordinating study meetings, supervising the study, monitoring data safety, and overseeing quality control.

### Study design

The study is designed as a prospective, randomized, controlled, single-center clinical trial. Patient enrollment started in April 2017, and the last patient is expected to be included in the study in April 2020. The ethics committee of the Shanghai General Hospital Institutional Review Board has granted ethics approval for this study (6 April 2017, Approval Number 2017 [16]).

### Study population/participants and recruitment

Recruitment will be carried out by responsible ophthalmic surgeons with a minimum of 10 years of vitrectomy experience in the Department of Ophthalmology, Shanghai General Hospital. Screening is done on day − 7~ 0 prior to the treatment in order to ensure that patients fulfill the inclusion criteria. Patients will attend an informational meeting, where they will be informed about the study purpose, process, and possible profits and risks. Patients fulfilling the study criteria who have signed the informed consent form will start treatment in accordance with the standard routines of the trial site. The informed consent will be obtained by the investigators. During the trial, the investigators will continue to provide additional health care or compensation for participants’ health care needs that arise as a direct consequence of trial participation.

### Eligibility criteria

#### Inclusion criteria

The inclusion criteria are as follows:

- Prior written informed consent must be obtained before any assessment is carried out.
- Participants are male or female Chinese patients who are more than 18 years and less than 75 years of age.
- Visual impairment is caused by a macular hole associated with retinal detachment secondary to high myopia.
- The axial length ≥ 26 mm, the refractive error ≥ − 6.0 D.

#### Exclusion criteria

The exclusion criteria are as follows:

- Failure to comply with research or follow-up procedures
- Diabetes with uncontrolled blood glucose (defined as fasting plasma glucose more than 7.0 mmol/L or blood glucose more than 11.1 mmol/L 2 h postprandial) and/or with diabetic retinopathy
- Poor control of blood pressure in hypertensive patients (defined as blood pressure > 150/95 mmHg, including antihypertensive medication)
- Surgical contraindication due to other local or systemic conditions at screening or baseline
- Any active ocular or periocular infection or inflammation (e.g., blepharitis, conjunctivitis, keratitis, scleritis, uveitis, endophthalmitis) at screening or baseline
- Uncontrolled glaucoma at screening or baseline (intraocular pressure (IOP) ≥ 30 mmHg when receiving medical treatment or as judged by the researchers)
- The presence of iris neovascularization or neovascular glaucoma at screening or baseline
- Ocular diseases which may interfere with the study results at screening or baseline, including severe vitreous hemorrhage, peripheral retinal hole, proliferative diabetic retinopathy, proliferative vitreoretinopathy (≥ level C), choroidal detachment
- Other causes which may result in macular hole-associated retinal detachment at screening or baseline, except high myopia
- Patients who have previously undergone scleral buckling surgery
- Patients with current or planned medication known to have toxic effects on the lens, retina or optic nerve, including hydroxychloroquine, chloroquine, tamoxifen, phenothiazine, and ethambutol
- Laboratory abnormalities, such as alanine aminotransferase (ALT), aspartate transaminase (AST), total bilirubin (TB), γ-glutamyl transpeptidase (GGT), or lactic dehydrogenase (LDH), exceeding the normal limit by more than two times, and creatinine (CREA) or blood urea nitrogen exceeding 1.2 times the normal limit
- Abnormal coagulation function (defined as a longer than normal prothrombin time by 3 s or more, more than 1.5 of the international standard ratio (INR), or an activated partial thromboplastin time 10 s or longer than the upper limit of normal time)
- Patients who participated in any clinical study of medication within 3 months prior to screening (excluding vitamins and minerals)

#### Exit criteria

The exit criteria include the following:

- Due to adverse events, especially severe adverse events, the researchers consider withdrawal of patient(s) based on ethical and safety concerns.
- Drop out.
- Patients who voluntarily withdraw their informed consent.
- Serious violation of the study protocol for reasons of the subjects or investigators.
- Other reasons that the researchers believe are acceptable for quitting the study.

### Randomization

Informed consent will be obtained from each participant before patient enrollment in the study. Patients who meet all the inclusion criteria and none of the exclusion criteria will be consecutively included and randomized into one of the two study arms by the study statistician, who has no involvement in the enrollment, assignment, or assessment of patients, on a random allocation sequence generated by Software Stata 7.0. The randomization list is kept strictly confidential. Allocation concealment is ensured with the use of sequentially numbered, identical, opaque, sealed envelopes. The intervention will be assigned by a nurse, who has also no involvement in the enrollment or assessment of patients, who will open the sealed envelope during the visit before surgery.

- *Group 1*. The patients in Group 1 are treated by the surgical method of standard 3-port 23-gauge pars plana vitrectomy plus ILM peeling plus air-fluid exchange plus silicone oil infusion.
- *Group 2*. The patients in Group 2 are treated by the surgical method of standard 3-port 23-gauge pars plana vitrectomy plus ILM peeling plus inverted ILM insertion plus air-fluid exchange.

Randomization will be stratified by the extent of retinal detachment: type 1 (within the arcade area) vs. type 2 (beyond the arcade area).

### Blinding

In this study, the third-party independent evaluation method is used to evaluate the outcomes of the study. The outcome analyzer and the study statistician are in the masking state, and the patients and the surgeons are in the non-masking state.

### General procedure and monitoring

#### Data collection and management

**Fig. 1:**
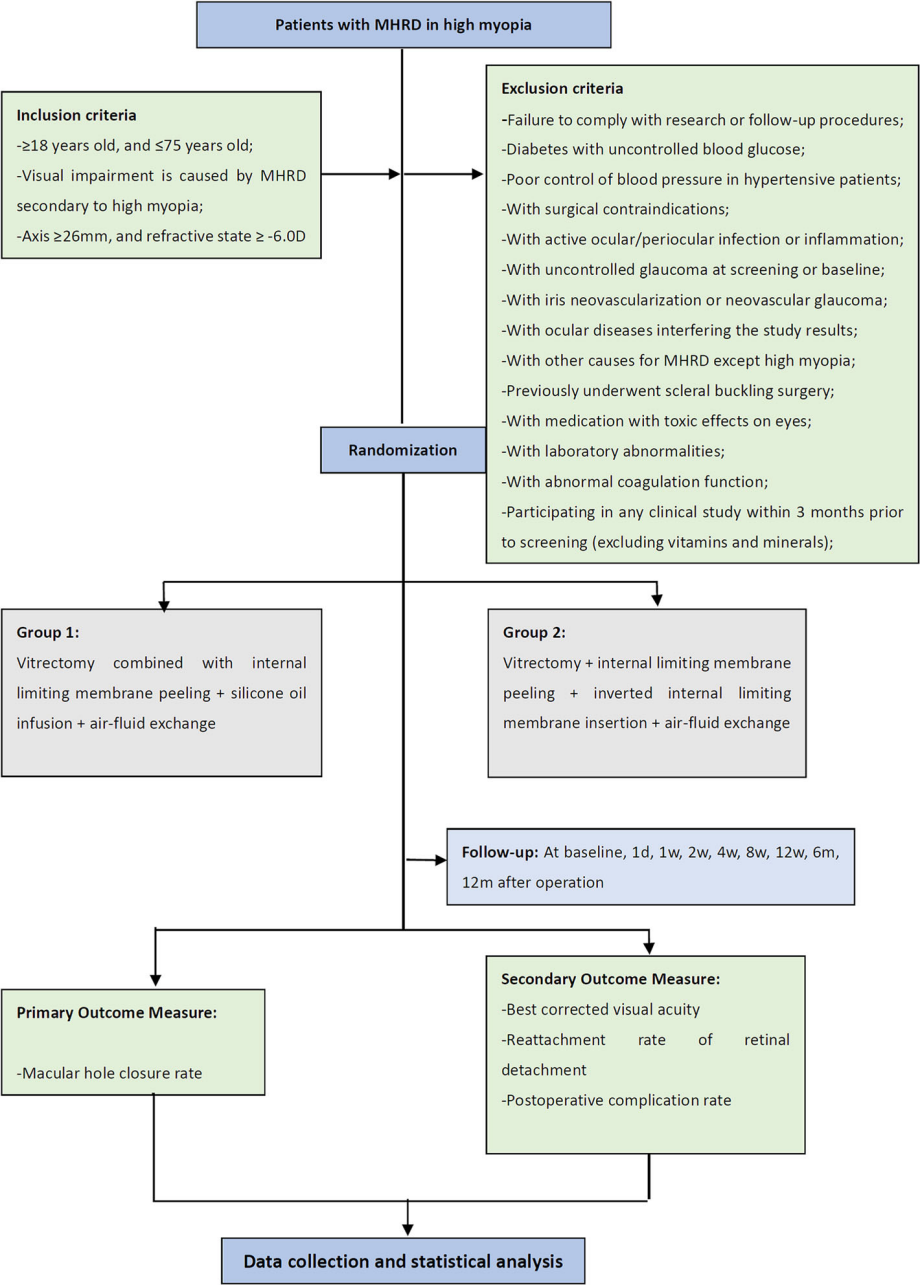
Study design

Treatment-related data are collected at baseline and on day 0 (D0). Follow-up data according to the study protocol will be followed from D1 to month 12 (M12). Data collection begins on the day a participant signs the informed consent and continues until the termination of the trial or until the participant withdraws from the trial at any time for any reason. If participants discontinue or deviate from the study protocols, the investigators will make best efforts to keep all missing data to a minimum. All original data are kept in chronological order for verification. Original data are timely transferred to a paper-based case report form (CRF) and an electronic database system located in a guarded facility at the trial site. Access to the study data is restricted. The PI will have the access to the final dataset. All data files have a complete audit trail. An independent steering committee will monitor and examine abidance with the study protocol (Fig. 1 and Table 1).

**Table 1:** General procedure and monitoring process

Period	Screening period	Randomization period	Follow-up period^a^								
Project	Baseline	Treatment	V1	V2	V3	V4	V5	V6	V7	Termination	Unscheduled visit
Visiting time	–7~0 d	0 d	1 d	1 w	2 w	4 w	8 w	12 w	6 m	12 m	–
Informed consent	×										
Inclusion and exclusion criteria	×	×^b^									
Demographic data	×										
Chief complaint and current medical history	×										
Medical history	×										
Previous surgery/trauma history and ocular surgery history	×										
Diagnosis	×										
Vital signs^c^	×		×	×	×	×	×	×	×	×	×
General physical examination	×		×	×	×	×	×	×	×	×	×
Slit lamp examination^d^ (binocular)	×										
Slit lamp examination^e^ (study eye)			×	×	×	×	×	×	×	×	×
Electrocardiograph (ECG) and chest radiography^f^	×										
Laboratory examination^g^	×										
Auxiliary examinations^h^	×		×	×	×	×	×	×	×	×	×
Low Vision Quality-of-Life Questionnaire^i^	×						×	×	×	×	
History change		×									
Randomization		×									
Surgical treatment		×									
Subject files	×										
Adverse events^j^		×	×	×	×	×	×	×	×	×	×
Previous/combined medication^k^	×	×	×	×	×	×	×	×	×	×	×
Quit in advance			×	×	×	×	×	×	×	×	
Unscheduled visits											×

Remarks:

*V* visit, *d* day, *w* week, *m* month, *BCVA* best corrected visual acuity, *INR* international standard ratio, *OU* oculus uterque (both eyes), *OCT* optical coherence tomography

^a^Visit window: visit 2 to visit 6; each visit has a window period of ± 3 days; visit 7 and end visit allow a window period of ± 7 days

^b^If both the eyes meet the requirements in the screening, the eye with the poorer BCVA should be selected as the study eye, unless for medical reasons, the researchers believe that the other eye is more suitable for the research. The study eye will undergo surgical treatment according to the protocol. If the BCVA damage of the contralateral eye is caused by macular hole associated with retinal detachment secondary to high myopia, according to the judgment of the researchers, the contralateral eye can also be recommended for surgical treatment. The contralateral eye is labeled as the contralateral eye for treatment. The treatment of bilateral eyes should not be performed on the same day

^c^Vital signs, including blood pressure, pulse, respiration, and armpit temperature, should be measured after patient has been sitting for 5 min

^d^Slit lamp examination: use “Slit Lamp Examination Form-I” (Additional file 2: Attachment 1). Accept the examination results carried out before the informed consent signature during the same hospitalization

^e^Slit lamp examination: use “Slit Lamp Examination Form-II” (Additional file 2: Attachment 2)

^f^ECG, chest radiography: accept the examination results carried out before the informed consent signature during the same hospitalization

^g^Laboratory examination includes routine blood test, blood biochemistry, routine blood coagulation, hepatitis B quantification, hepatitis C virus (HCV) antibody, *Treponema pallidum* particle agglutination assay (TPPA) plus rapid plasma reagin assay (RPR), and human immune deficiency virus (HIV) antibody. Accept the examination results carried out before the informed consent signature during the same hospitalization

①Routine blood test: white blood cells, neutrophils, lymphocytes, neutrophil ratio, lymphocyte ratio, red blood cells, hemoglobin, platelets

②Blood biochemistry: alanine aminotransferase, aspartate aminotransferase, alkaline phosphatase, lactate dehydrogenase, γ-glutamyl transferase, total bilirubin, direct bilirubin, total protein, albumin, urea, creatinine, uric acid, glucose, triglycerides, total cholesterol, potassium, sodium, chloride, calcium, phosphorus

③Routine blood coagulation: prothrombin time, activated partial thromboplastin time, thrombin time, fibrinogen, prothrombin time-INR

^h^Auxiliary examinations:

①Auxiliary examinations in the screening period include BCVA OU, IOP OU, OCT of study eye, wide angle color photo (CP) of study eye, multifocal electroretinogram of study eye, microperimetry and visual field analysis of study eye, ocular axis measurement of study eye, B-scan ultrasonography of study eye. Accept the examination results carried out before the informed consent signature during the same hospitalization

②Auxiliary examinations at V1 and V2 include BCVA OU, IOP OU, OCT of study eye, wide angle CP of study ocular axial measurement of study eye

③ Auxiliary examinations at V3 include BCVA OU, IOP OU, OCT of study eye, wide angle CP of study eye, B-scan ultrasonography of study eye

④Auxiliary examinations at V4 include BCVA OU, IOP OU, OCT of study eye, wide angle CP of study eye

⑤Auxiliary examinations at V5, V6, V7, end visits, and unscheduled visits include BCVA OU, IOP OU, OCT of study eye, wide angle CP of study eye, multifocal electroretinogram of study eye, microperimetry and visual field analysis of study eye, and B-scan ultrasonography of study eye

^i^The Low Vision Quality-of-Life Questionnaire: subjects were asked to self-rate according to the Low Vision Quality-of-Life Questionnaire (Additional file 2: Attachment 3)

^j^Adverse events (AEs): adverse event information is collected from the time the subjects sign informed consent until the end of the study

^k^Previous/combined medication: collection of relevant information from the time the subjects sign informed consent until the end of the study, including all the medication used for the treatment of AEs or SAEs

### Study outcome variables

#### Primary outcome

The primary outcome is macular hole closure rate. A fundus examination combined with optical coherence tomography (OCT) will be performed 3 months after the initial surgery. Macular hole closure is defined as absence of neurosensory defect over the fovea in the OCT images [12, 13, 15].

#### Secondary outcomes

The secondary outcomes are:

- Best corrected visual acuity (BCVA, assessed with LogMAR chart) 6 months after the surgery
- Reattachment rate of retinal detachment. Fundus examination combined with B-scan ultrasonography and OCT are performed to observe the reattachment rate of retinal detachment within 12 months after the operation. (The reattachment rate assessment is performed 12 months after the first surgery among the patients with air tamponade. The reattachment rate assessment is performed 12 months after the first surgery among the patients with silicone oil tamponade, and the silicone oil removal is performed 6 months after the previous surgery)
- Postoperative complication rate

### Statistical methods

#### Sample size

The sample size of this superiority trial was estimated based on the literature and our own unpublished data. According to the references, professional judgment, and our preliminary experiment results, the macular hole closure rate is expected to be 25% in the control group (Group 1), while in the experimental group (Group 2), macular hole closure rate should be 65%. The significance level α is 0.05, and the test performance (1 – β) is 0.80. The software NCSS PASS 14 is used, and each group has 17 cases with a loss rate of 10%. The final sample size is 38.

#### Data analysis

Normally distributed variables will be expressed as their mean and standard deviation (SD) and non-normally distributed variables will be expressed as their median and interquartile range; categorical variables will be expressed as the sample size number plus percentage (*n*, %). In test groups of continuous normally distributed variables, the Student *t* test will be used; the Mann–Whitney *U* test will be used for continuous non-normally distributed data. Categorical variables will be compared with the χ^2^ test or Fisher’s exact test or, when appropriate, as the relative risk. Statistical analysis will be conducted on an intention-to-treat (ITT) basis. Multivariable analysis will be conducted by logistic regression and generalized mixed linear regression model to take into account any possible confounding covariate adjustments necessary, and also to consider within-center variability. A *p* value of < 0.05 will be considered as statistically significant.

#### Populations for evaluation and missing data management

All evaluations, in particular the evaluation of the primary outcome measure, will be made on the basis of all randomized patients, regardless of whether or not they adhered to the treatment protocol or provided complete data sets. In particular, these latter patients are those:

- Who discontinued the clinical trial; they will be evaluated as if they had complied with it
- Whose planned examinations were not carried out within the planned time frame; they will still be taken into consideration in the analysis

Patients who withdraw their consent to use their personal data for statistical analyses will be excluded from the analysis.

Missing reports of individual responses on the Low Vision Quality-of-Life Questionnaire will be replaced by simple imputation according to the recommendations of the test manual.

The reason for the missing data will be analyzed, and the data missing at random will be handled with multiple imputation and model-based approaches, such as mixed models or weighted generalized estimating equations (GEEs) for repeatedly measured outcomes. Sensitivity analysis will be performed to examine the robustness of the results to assumptions made in the complete case analysis.

### Adverse events

An adverse event (AE) refers to any untoward event that occurs during the clinical study but does not necessarily have a causal relationship with the surgical treatment. Safety evaluation is carried out from the point at which the signature of the informed consent is obtained until the end of the study or until patient withdrawal from the trial, according to management requirements. Adverse events or serious adverse events should be reported.

A serious adverse event (SAE) refers to an event that causes hospitalization, prolonged hospitalization, disability, incapacity, life-threatening illness or death, or congenital malformation during the clinical trial.

During the study, all AEs are recorded. Records include the name of the AE (using standard medical terminology), the date of the AE occurrence, and disappearance/stability, severity, impact on the surgery, relationship with the surgery, treatment measures, and outcomes. If an SAE occurs, researchers fill in an SAE Report . The report is signed and dated and reported to the ethics committee and the clinical research center of Shanghai General Hospital within 24 h.

### Quality control

All surgeons and analyzers will be required to undergo special training prior to the trial to guarantee consistent practice. The training program will include diagnosis, inclusion/exclusion/exit criteria, surgery techniques, follow-up procedures, and completion of CRFs. The trial will be monitored by quality assurance personnel from the clinical research center in Shanghai General Hospital, who will be independent from the study team, and an independent steering committee. There will be periodic monitoring to guarantee accuracy and quality throughout the study period. The essential documents (consent information, enrollment, protocol deviations, number and proportion of missed visits, and losses to follow-up) will be monitored and checked for accuracy and completeness by the monitors.

### Confidentiality

The relevant regulations of the data protection legislation will be entirely fulfilled. All appropriate and necessary precautionary measures will be met in order to perpetuate the confidentiality of medical data and personal information. The data safety will be monitored by quality assurance personnel from the clinical research center in Shanghai General Hospital, who will be independent from the study team, and also an independent steering committee.

### Regulatory and ethical approval

The ethics committee of the Shanghai General Hospital Institutional Review Board has granted ethics approval for this study (6 April 2017, Approval Number 2017 [16]).

In case of a necessary protocol amendment, the amendment will be submitted to the ethics committee and the quality assurance personnel from the clinical research center in Shanghai General Hospital, who will be independent from the study team. Competent authority and implementation will be performed after approval. Due to the study design (one-centered, investigator-initiated trial) and the close contact between the study teams and site, a separate communication plan is not necessary.

## Discussion

High myopia-associated MHRD, a challenging disease for vitreoretinal surgeons, usually causes severe visual impairment and greatly reduces the quality of a patient’s life. The main treatment for MHRD currently is vitrectomy combined with ILM peeling plus expansive gas or silicone oil tamponade, which has a high rate of postoperative complications. Thus, new surgical methods with more effectiveness and less pain are required during clinical practice.

This study aims to evaluate the efficacy and safety of inverted ILM insertion combined with air tamponade in the treatment of high myopia-associated MHRD, and also to compare the treatment efficacy with that of the conventional surgical approach. If it is found to be significantly effective and safe, this trial will provide a novel surgical method for MHRD in high myopia Additional files 1 and 2.

### Trial status

At the time of initial manuscript submission, recruitment had already started (April 2017), but it has not been completed. The last patient is expected to be included in the study in April 2020. The manuscript reports protocol version 3.0 (11 November 2017).

## Additional files

Additional file 1: SPIRIT 2013 checklist. (DOC 127 kb)

Additional file 2: Attachments 1, 2 and 3 [Slit Lamp Examination Form (right eye)]. (DOCX 35 kb)

## Abbreviations

AE: Adverse event
ALT: Alanine aminotransferase
AST: Aspartate transaminase
BCVA: Best corrected visual acuity
CP: Color photo
CREA: Creatinine
ECG: Electrocardiograph
GGT: γ-glutamyl transpeptidase
HCV: Hepatitis C virus
HIV: Human immune deficiency virus
ILM: Internal limiting membrane
INR: Iinternational standard ratio
IOP: Intraocular pressure
ITT: Intention-to-treat
LDH: Lactic dehydrogenase
MH: Macular hole
MHRD: Macular hole retinal detachment
OCT: Optical coherence tomography
RPR: Rapid plasma reagin assay
SAE: Serious adverse event
TB: Total bilirubin
TPPA: *Treponema pallidum* particle agglutination assay
