# Supplementary material for: Inverted internal limiting membrane insertion combined with air tamponade in the treatment of macular hole retinal detachment in high myopia: study protocol for a randomized controlled clinical trial
Source: Trials. 2018 Aug 30;19:469. doi: 10.1186/s13063-018-2833-y (PMC6117933; doi:10.1186/s13063-018-2833-y)
Supplement: Supplementary file 2 — Attachments 1, 2 and 3 [Slit Lamp Examination Form (right eye)]. (DOCX 35 kb) [file 13063_2018_2833_MOESM2_ESM.docx]

**Attachment 1**

**【Slit Lamp Examination Form (right eye)】-I**

| Whether to have slit lamp examination (right eye)?：_1_□Yes→Please record as follows，_0_□No | |
| --- | --- |
| Other lesions of eyelid | _0_□No  _1_□Yes，Please describe：_________________________________ |
| 1. Conjunctiva (right eye) | |
| Congestion | _0_□Normal. It may appear white or reddish pink, without peripheral congestion. The conjunctival or bulbar conjunctival vessels are easily observed  _1_□Slight redness, reddish color, mainly confined to the palpebral conjunctiva or bulbar conjunctiva  _2_□Mild redness, reddish color, mainly confined to the conjunctiva or bulbar conjunctiva  _3_□Moderate, palpebral conjunctiva or bulbar conjunctiva is bright red  _4_□Severe, palpebral conjunctiva or bulbar conjunctiva is deep diffuse bright red |
| Edema | _0_□Normal, no swelling  _1_□Slight, beyond normal and regional  _2_□Mild, mild conjunctival swelling, beyond normal, regional  _3_□Moderate, conjunctival swelling moderately  _4_□Severe, large conjunctival swelling |
| Subconjunctival hemorrhage | _0_□Nil, no bleeding  _1_□Slight, flat and less than 1 quadrant  _2_□Mild, bulging, and 1 quadrant, or flat and more than 1 quadrants  _3_□Moderate, bulging and >1 quadrant, but less than 2 quadrants  _4_□Severe, bulging and more than 2 quadrants |
| Other conjunctival lesions | _0_□No  _1_□Yes，Please describe：_________________________________ |
| 2. Cornea (right eye) | |
| Edema | _0_□Nil, transparent and clear  _1_□Slight, micro and local epithelial haze  _2_□Mild, turbid vitreous appearance and may contain tiny droplets  _3_□Moderate, turbid vitreous appearance with a large number of vacuoles  _4_□Severe, bullous and / or stromal edema, localized or diffuse, with or without matrix streaks |
| Other corneal lesions | _0_□No  _1_□Yes，Please describe：_________________________________ |

**【Slit Lamp Examination Form (right eye)】-I**

| 3. Anterior chamber (right eye) | |
| --- | --- |
| Cell number | _0_□Nil, no cells  _1_□Slight, 1-5 cells  _2_□Mild, 6-25 cells  _3_□Moderate, 26-50 cells  _4_□Severe, the number of cells is too large to count |
| Anterior chamber flare | _0_□Nil, no Tyndall effect  _1_□Slight, almost cannot distinguish the Tyndall effect  _2_□Mild, slight brightness  _3_□Moderate, strong brightness  _4_□Severe, very strong and aqueous humor is white or milky |
| Other lesions | _0_□No  _1_□Yes，Please describe：_________________________________ |
| 4. Iris / pupil (right eye) | |
| Iris / pupil (right eye) | Clinically significant abnormality：_0_□No_1_□Yes，Please describe：_____________ |
| 5. Lens (right eye) | |
| Lens (right eye) | _1_□Complete  _2_□Artificial  _3_□Nil |
| Other lesions | _0_□No  _1_□Yes，Please describe：_________________________________ |
| Transparency | |
| Cortex | _0_□Nil  _1_□There are turbidity, but below standard pictures 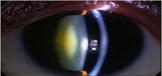  _2_□There are turbidity, and the severity of the standard picture is the same or more serious 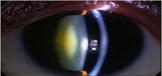  _3_□Not applicable |
| Nucleus | _0_□Nil  _1_□Turbid, but below standard pictures 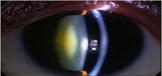  _2_□Turbid, the same or more serious than standard pictures 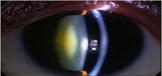  _3_□Not applicable |
| Posterior capsule | _0_□Nil  _1_□Turbid, but below standard pictures 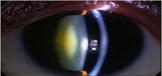  _2_□Turbid, the same or more serious than standard pictures 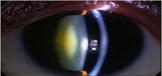  _3_□Not applicable |

**【Slit Lamp Examination Form (right eye)】-I**

| 6. Vitreum (right eye) | |
| --- | --- |
| Anterior vitreous cell count | _0_□Nil, no cells  _1_□Slight, 1-10 cells  _2_□Mild, 11-30 cells  _3_□Moderate, 30-50 cells  _4_□Severe, >50 cells |
| Other lesions | _0_□No  _1_□Yes，Please describe：_________________________________ |
| Vitreous / anterior retinal hemorrhage | _0_□Nil  _1_□Slight  _2_□Mild  _3_□Moderate  _4_□Severe |
| 7. Macular and retina (right eye) | |
| Macular hole | _0_□Nil  _1_□Hole diameter≤1/3PD  _2_□Hole diameter＞1/3PD, and≤1/2PD  _3_□Hole diameter＞1/2PD, and≤1PD  _4_□Hole diameter＞1PD |
| Retinal detachment | _0_□Nil  _1_□Yes，Please describe：  _1_□Within the arcade  _2_□Beyond the arcade, and within the equator  _3_□Beyond the equator |
| Peripheral retinal split holes | _0_□No  _1_□Yes，Please describe：_________________________________ |
| Posterior scleral staphyloma | _0_□No  _1_□Yes，Please describe：_________________________________ |
| Other lesions | _0_□No  _1_□Yes，Please describe with words or pictures：_________________________________ |

**Attachment 1**

**【Slit Lamp Examination Form (left eye)】-I**

| Whether to have slit lamp examination (left eye)?：_1_□Yes→Please record as follows，_0_□No | |
| --- | --- |
| Other lesions of eyelid | _0_□No  _1_□Yes，Please describe：_________________________________ |
| 1. Conjunctiva (right eye) | |
| Congestion | _0_□Normal. It may appear white or reddish pink, without peripheral congestion. The conjunctival or bulbar conjunctival vessels are easily observed  _1_□Slight redness, reddish color, mainly confined to the palpebral conjunctiva or bulbar conjunctiva  _2_□Mild redness, reddish color, mainly confined to the conjunctiva or bulbar conjunctiva  _3_□Moderate, palpebral conjunctiva or bulbar conjunctiva is bright red  _4_□Severe, palpebral conjunctiva or bulbar conjunctiva is deep diffuse bright red |
| Edema | _0_□Normal, no swelling  _1_□Slight, beyond normal and regional  _2_□Mild, mild conjunctival swelling, beyond normal, regional  _3_□Moderate, conjunctival swelling moderately  _4_□Severe, large conjunctival swelling |
| Subconjunctival hemorrhage | _0_□Nil, no bleeding  _1_□Slight, flat and less than 1 quadrant  _2_□Mild, bulging, and 1 quadrant, or flat and more than 1 quadrants  _3_□Moderate, bulging and >1 quadrant, but less than 2 quadrants  _4_□Severe, bulging and more than 2 quadrants |
| Other conjunctival lesions | _0_□No  _1_□Yes，Please describe：_________________________________ |
| 2. Cornea (left eye) | |
| Edema | _0_□Nil, transparent and clear  _1_□Slight, micro and local epithelial haze  _2_□Mild, turbid vitreous appearance and may contain tiny droplets  _3_□Moderate, turbid vitreous appearance with a large number of vacuoles  _4_□Severe, bullous and / or stromal edema, localized or diffuse, with or without matrix streaks |
| Other corneal lesions | _0_□No  _1_□Yes，Please describe：_________________________________ |

**【Slit Lamp Examination Form (left eye)】-I**

| 3. Anterior chamber (left eye) | |
| --- | --- |
| Cell number | _0_□Nil, no cells  _1_□Slight, 1-5 cells  _2_□Mild, 6-25 cells  _3_□Moderate, 26-50 cells  _4_□Severe, the number of cells is too large to count |
| Anterior chamber flare | _0_□Nil, no Tyndall effect  _1_□Slight, almost cannot distinguish the Tyndall effect  _2_□Mild, slight brightness  _3_□Moderate, strong brightness  _4_□Severe, very strong and aqueous humor is white or milky |
| Other lesions | _0_□No  _1_□Yes，Please describe：_________________________________ |
| 4. Iris / pupil (left eye) | |
| Iris / pupil (right eye) | Clinically significant abnormality：_0_□No_1_□Yes，Please describe：_____________ |
| 5. Lens (left eye) | |
| Lens (right eye) | _1_□Complete  _2_□Artificial  _3_□Nil |
| Other lesions | _0_□No  _1_□Yes，Please describe：_________________________________ |
| Transparency | |
| Cortex | _0_□Nil  _1_□There are turbidity, but below standard pictures 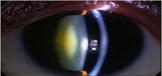  _2_□There are turbidity, and the severity of the standard picture is the same or more serious 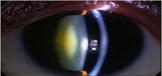  _3_□Not applicable |
| Nucleus | _0_□Nil  _1_□Turbid, but below standard pictures 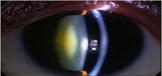  _2_□Turbid, the same or more serious than standard pictures 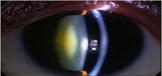  _3_□Not applicable |
| Posterior capsule | _0_□Nil  _1_□Turbid, but below standard pictures 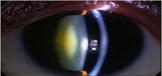  _2_□Turbid, the same or more serious than standard pictures 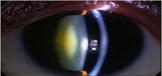  _3_□Not applicable |

**【Slit Lamp Examination Form (left eye)】-I**

| 6. Vitreum (left eye) | |
| --- | --- |
| Anterior vitreous cell count | _0_□Nil, no cells  _1_□Slight, 1-10 cells  _2_□Mild, 11-30 cells  _3_□Moderate, 30-50 cells  _4_□Severe, >50 cells |
| Other lesions | _0_□No  _1_□Yes，Please describe：_________________________________ |
| Vitreous / anterior retinal hemorrhage | _0_□Nil  _1_□Slight  _2_□Mild  _3_□Moderate  _4_□Severe |
| 7. Macular and retina (left eye) | |
| Macular hole | _0_□Nil  _1_□Hole diameter≤1/3PD  _2_□Hole diameter＞1/3PD, and≤1/2PD  _3_□Hole diameter＞1/2PD, and≤1PD  _4_□Hole diameter＞1PD |
| Retinal detachment | _0_□Nil  _1_□Yes，Please describe：  _1_□Within the arcade  _2_□Beyond the arcade, and within the equator  _3_□Beyond the equator |
| Peripheral retinal split holes | _0_□No  _1_□Yes，Please describe：_________________________________ |
| Posterior scleral staphyloma | _0_□No  _1_□Yes，Please describe：_________________________________ |
| Other lesions | _0_□No  _1_□Yes，Please describe with words or pictures：_________________________________ |

**Attachment 2**

**【Slit Lamp Examination Form (study eye)】-II**

| Whether to have slit lamp examination (study eye)?：_1_□Yes→Please record as follows，_0_□No | |
| --- | --- |
| Other lesions of eyelid | _0_□No  _1_□Yes，Please describe：_________________________________ |
| 1. Conjunctiva (study eye) | |
| Congestion | _0_□Normal. It may appear white or reddish pink, without peripheral congestion. The conjunctival or bulbar conjunctival vessels are easily observed  _1_□Slight redness, reddish color, mainly confined to the palpebral conjunctiva or bulbar conjunctiva  _2_□Mild redness, reddish color, mainly confined to the conjunctiva or bulbar conjunctiva  _3_□Moderate, palpebral conjunctiva or bulbar conjunctiva is bright red  _4_□Severe, palpebral conjunctiva or bulbar conjunctiva is deep diffuse bright red |
| Edema | _0_□Normal, no swelling  _1_□Slight, beyond normal and regional  _2_□Mild, mild conjunctival swelling, beyond normal, regional  _3_□Moderate, conjunctival swelling moderately  _4_□Severe, large conjunctival swelling |
| Subconjunctival hemorrhage | _0_□Nil, no bleeding  _1_□Slight, flat and less than 1 quadrant  _2_□Mild, bulging, and 1 quadrant, or flat and more than 1 quadrants  _3_□Moderate, bulging and >1 quadrant, but less than 2 quadrants  _4_□Severe, bulging and more than 2 quadrants |
| Other conjunctival lesions | _0_□No  _1_□Yes，Please describe：_________________________________ |
| 2. Cornea (study eye) | |
| Edema | _0_□Nil, transparent and clear  _1_□Slight, micro and local epithelial haze  _2_□Mild, turbid vitreous appearance and may contain tiny droplets  _3_□Moderate, turbid vitreous appearance with a large number of vacuoles  _4_□Severe, bullous and / or stromal edema, localized or diffuse, with or without matrix streaks |
| Other corneal lesions | _0_□No  _1_□Yes，Please describe：_________________________________ |

**【Slit Lamp Examination Form (study eye)】-II**

| 3. Anterior chamber (study eye) | |
| --- | --- |
| Cell number | _0_□Nil, no cells  _1_□Slight, 1-5 cells  _2_□Mild, 6-25 cells  _3_□Moderate, 26-50 cells  _4_□Severe, the number of cells is too large to count |
| Anterior chamber flare | _0_□Nil, no Tyndall effect  _1_□Slight, almost cannot distinguish the Tyndall effect  _2_□Mild, slight brightness  _3_□Moderate, strong brightness  _4_□Severe, very strong and aqueous humor is white or milky |
| Other lesions | _0_□No  _1_□Yes，Please describe：_________________________________ |
| 4. Iris / pupil (study eye) | |
| Iris / pupil (study eye) | Clinically significant abnormality：_0_□No_1_□Yes，Please describe：_____________ |
| 5. Lens (studyeye) | |
| Lens (study eye) | _1_□Complete  _2_□Artificial  _3_□Nil |
| Other lesions | _0_□No  _1_□Yes，Please describe：_________________________________ |
| Transparency | |
| Cortex | _0_□Nil  _1_□There are turbidity, but below standard pictures 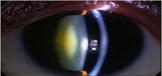  _2_□There are turbidity, and the severity of the standard picture is the same or more serious 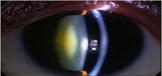  _3_□Not applicable |
| Nucleus | _0_□Nil  _1_□Turbid, but below standard pictures 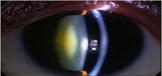  _2_□Turbid, the same or more serious than standard pictures 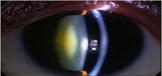  _3_□Not applicable |
| Posterior capsule | _0_□Nil  _1_□Turbid, but below standard pictures 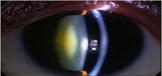  _2_□Turbid, the same or more serious than standard pictures 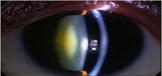  _3_□Not applicable |

**【Slit Lamp Examination (study eye)】-II**

| 6. Vitreum (study eye) | |
| --- | --- |
| Anterior vitreous cell count | _0_□Nil, no cells  _1_□Slight, 1-10 cells  _2_□Mild, 11-30 cells  _3_□Moderate, 30-50 cells  _4_□Severe, >50 cells |
| Other lesions | _0_□No  _1_□Yes，Please describe：_________________________________ |
| Vitreous / anterior retinal hemorrhage | _0_□Nil  _1_□Slight  _2_□Mild  _3_□Moderate  _4_□Severe |
| Air volume in vitreous cavity | _0_□Nil  _1_□≤30%  _2_□＞30%，and≤50%  _3_□＞50%，and≤70%  _4_□＞70%  _5_□Not applicable |
| Silicone oil volume in vitreous cavity | _0_□Nil  _1_□＞50%，and≤70%  _2_□＞70%，and≤90%  _3_□＞90%  _4_□Not applicable |
| 7. Macular and retina (research eye) | |
| Macular hole | _0_□Nil  _1_□Hole diameter≤1/3PD  _2_□Hole diameter＞1/3PD, and≤1/2PD  _3_□Hole diameter＞1/2PD, and≤1PD  _4_□Hole diameter＞1PD |
| Retinal detachment | _0_□Nil  _1_□Yes，Please describe：  _1_□Within the arcade  _2_□beyond the arcade, and within the equator  _3_□Beyond the equator |
| Peripheral retinal split holes | _0_□No  _1_□Yes，Please describe：_________________________________ |
| Posterior scleral staphyloma | _0_□No  _1_□Yes，Please describe：_________________________________ |
| Other lesions | _0_□No  _1_□Yes，Please describe with words or pictures：_________________________________ |

**Attachment 3**

**Low vision quality of life questionnaire**

5=Nil；4-2=Moderate；1=Severe；x=Unable to do。

How well do you know about your current vision?：5=Much，1=Little

| 1. Because of your vision, in the following circumstances, do you feel difficult?. | |
| --- | --- |
| 1. Feel tired (for example, only after a short time using eyes)  usingeye use) | □5 □4 □3 □2 □1□x □Others |
| 2. At home at night | □5 □4 □3 □2 □1□x □Others |
| 3. Look at things in the right amount of light | □5 □4 □3 □2 □1□x □Others |
| 4. Feel the light glare (such as car lamp or sun make you dazzle) | □5 □4 □3 □2 □1□x □Others |
| 5. Look at road signs | □5 □4 □3 □2 □1□x □Others |
| 6. Watch TV (or enjoy pictures) | □5 □4 □3 □2 □1□x □Others |
| 7. Look at moving objects (such as cars on the road) | □5 □4 □3 □2 □1□x □Others |
| 8. Determine the distance or depth of objects | □5 □4 □3 □2 □1□x □Others |
| 9. See the stairs or railings | □5 □4 □3 □2 □1□x □Others |
| 10. Walk outdoors (e.g., on an uneven sidewalk) | □5 □4 □3 □2 □1□x □Others |
| 11. Cross the street when there is vehicles | □5 □4 □3 □2 □1□x □Others |
| 12. Generally speaking | □5 □4 □3 □2 □1□x □Others |
| 2. Because of your vision, so you | |
| 1. Not satisfied with your current life | □5 □4 □3 □2 □1□x □Others |
| 2. Be upset about not being able to do some work | □5 □4 □3 □2 □1□x □Others |
| 3. Visiting relatives and friends is limited | □5 □4 □3 □2 □1□x □Others |
| 4..How well do you know about your current vision | □5 □4 □3 □2 □1□x □Others |
| 3. If you use visual aids or glasses, if there are difficulties when doing the following things | |
| 1.Identify uppercase fonts | □5 □4 □3 □2 □1□x □Others |
| 2. Read newspaper articles and books | □5 □4 □3 □2 □1□x □Others |
| 3. Read labels (for example, on a bottle or kit) | □5 □4 □3 □2 □1□x □Others |
| 4. Reading letters | □5 □4 □3 □2 □1□x □Others |
| 5. Use some tools (such as sutures or scissors) | □5 □4 □3 □2 □1□x □Others |
| 4. If you use visual aids or glasses, if there are difficulties when doing the following things | |
| 1. Watch the clock to know the time | □5 □4 □3 □2 □1□x □Others |
| 2. Sign or write cards | □5 □4 □3 □2 □1□x □Others |
| 3. Identify your own handwriting | □5 □4 □3 □2 □1□x □Others |
| 4. Do daily activities (such as housework) | □5 □4 □3 □2 □1□x □Others |
